# Correlation analysis of clinical, pathological, imaging and genetic features of ground-glass nodule featured lung adenocarcinomas between high-risk and non-high-risk individuals

**DOI:** 10.1186/s40001-023-01462-3

**Published:** 2023-11-04

**Authors:** Jing Ren, Yuan Wang, Chunrong Liu, Lan Yang, Xinlu Men, Zhixin Qiu

**Affiliations:** 1 Department of Pulmonary and Critical Care Medicine, West China Hospital, Sichuan University, Chengdu, 610041 Sichuan China; 2 The Integrated Care Management Center, West China Hospital, Sichuan University, Chengdu, 610041 Sichuan China; 3 Department of Pulmonary and Critical Care Medicine/Institute of Respiratory Health, Frontiers Science Center for Disease-related Molecular Network, West China Hospital, Sichuan University, Chengdu, 610041 Sichuan China; 4 Chinese Evidence-Based Medicine Center, West China Hospital, Sichuan University, Chengdu, 610041 Sichuan China; 5 Outpatient Department, West China Hospital, Sichuan University, Chengdu, 610041 Sichuan China; 6 Department of Pulmonary and Critical Care Medicine, State Key Laboratory of Respiratory Health and Multimorbidity, Precision Medicine Key Laboratory of Sichuan Province, West China Hospital, Sichuan University, Chengdu, 610041 Sichuan China

**Keywords:** Ground-glass nodule, Lung adenocarcinoma, High risk, Non-high risk, Gene

## Abstract

**Background:**

Early stage lung adenocarcinomas manifested as ground-glass nodules (GGNs) are increasingly being detected, but screening and diagnosis for GGN-featured lung adenocarcinomas in different risk populations reach no agreement.

**Objectives:**

To analyze the clinical, pathological, imaging and genetic features of GGN-featured lung adenocarcinomas on high-resolution computed tomography (HRCT) in different risk groups.

**Methods:**

Include patients with GGNs on HRCT surgically diagnosed as lung adenocarcinoma in the West China Hospital, Sichuan University from 2009 to 2021, and their clinical, pathological, imaging and gene sequencing data.

**Results:**

According to Chinese Expert Consensus on Screening and Management of Lung Cancer, 1,800 patients with GGN-featured lung adenocarcinoma, 545 males (incl. 269 smokers) and 1,255 females (incl. 16 smokers), were divided into high-risk (509) and non-high-risk (1,291) groups. Among them, 1,095 were detected via physical examination. The mean age at diagnosis was 54.78 (23–84) and the mean time from detection to diagnosis was 9.59 months. There were more males than females in the high-risk group [288 (56.58%) vs 221 (43.42%)], just the opposite in the non-high-risk group [1,034 (80.09%) vs 257 (19.91%)] (both *P* < 0.001). No statistical difference was found in GGN detection way (*P* > 0.05). The frequency of invasive adenocarcinoma was higher in the high-risk group, while those of precursor lesions and minimally invasive adenocarcinoma were higher in the non-high-risk group (all *P* < 0.001). The preoperative follow-up time in the non-high-risk group was shorter (*P* < 0.05). A total of 711 gene mutations were observed in 473 patients with a ratio of non-high-risk to high-risk of 494:217. The incidence of *EGFR* mutation was not statistically significant (*P* = 0.824), while those of *TP53* and *KRAS* mutations were higher in the high-risk group (*P* < 0.05).

**Conclusions:**

GGN-featured lung adenocarcinoma is dominated by non-high-risk female patients. Shorter preoperative follow-up in the non-high-risk group and no statistical difference in GGN detection way suggests the existing screening criteria for high-risk population may not suit GGN-featured lung cancer. In addition, the incidences of *KRAS* and *TP53* mutations are higher in the high-risk group.

**Supplementary Information:**

The online version contains supplementary material available at 10.1186/s40001-023-01462-3.

## Background

Lung cancer is the most common cancer and the most common cause of death from cancer worldwide [1, 2]. As early as 2011, a national lung cancer screening in the US suggested that, compared with radiography, screening with the use of low-dose computed tomography (LDCT) reduced mortality from lung cancer by 20% [3]. This is one of the greatest discoveries in lung cancer screening and clinical diagnosis trial so far. Since then, regular LDCT screening has been recommended to individuals at high risk for lung cancer for earlier screening, diagnosis and treatment, and guidelines for lung cancer screening have been formulated and improved gradually [4, 5]. In the Chinese Expert Consensus on Screening and Management of Lung Cancer [6], individuals at high risk for lung cancer are defined as the ages of 40 and older who have a 20 pack- (or 400 cigarette-)year smoking history and currently smoke or have quit within the past 15 years; have a history of environmental or occupational exposure (such as asbestos, beryllium, uranium, radon, etc.); are complicated with chronic obstructive pulmonary disease or diffuse pulmonary fibrosis, or have a history of pulmonary tuberculosis; or have a history of malignant tumor or a family history of lung cancer, especially those of first-degree relatives. Thanks to the breakthroughs in medical technology and the improvement of standard of living, the popularized chest CT facilitates the detection of early stage lung cancer manifesting as ground-glass nodules (GGNs) [7]. However, no consensus on the screening and diagnosis of GGN-featured lung cancers has been reached at home and abroad. Hence, by analyzing the correlation of the clinical, pathological, imaging and genetic features of GGN-featured lung adenocarcinoma between different risk populations, this study aims to provide a reference for the screening and diagnosis of early stage lung cancers.

## Materials and methods

### Subjects

From 2009 to 2021, patients with GGNs detected on HRCT images, surgically removed and pathologically confirmed as lung adenocarcinoma in West China Hospital of Sichuan University were collected. The following inclusion criteria were applied: (1) a ground-glass opacity on a CT scan with the maximum diameter of 30 mm; (2) the lesion with the longest diameter in case of multiple primary lung cancers; (3) imaging data of at least one lung CT scan; and (4) clear pathological results and complete clinical data. Exclusion criteria: (1) the density or diameter no up to the inclusion criteria; (2) incomplete clinical, pathological or imaging data; (3) complicated with severe pulmonary infection, interstitial lung disease, bronchiectasis, or cardiovascular disease; or 4) with a history of lung cancer.

### Clinical data

Gender, age, medical history, smoking history, family history of tumors, reason for GGN detection and time from follow-up to diagnosis, etc. were recorded. The medical history includes respiratory-related diseases and tumors. There are two reasons for GGN detection, namely, physical examination or screening due to some causes and symptoms.

### Imaging data

All the included cases had a HRCT scan with an optimum slice thickness of 1 mm in our hospital within 3 months before surgery. Images obtained with a mediastinal window and a lung window were reviewed and assessed by two chest subspecialists with more than 5 years of work experience at our picture archiving and communication systems (PACS) workstation. Imaging features include nodule size, location, density, lobulation, spiculation, vacuole, calcification, air bronchogram, vessel convergence, etc. (1) Size: the longest diameter (unit: mm) measured in the largest cross section of the nodule; (2) location: the superior and inferior lobes of the left lung, and the superior, middle, and inferior lobes of the right lung; and (3) density: pure ground-glass nodule (pGGN) and mixed ground-glass nodule (mGGN) [8].

### Pathology and gene mutation results

The pathology results of the resected tissue were used as the diagnostic criteria for GGNs. According to the 2021 WHO Classification of Lung Tumors [9], they were pathologically classified into three subtypes, namely, precursor lesions (incl. atypical adenomatous hyperplasia, AAH and adenocarcinoma in situ, AIS), minimally invasive adenocarcinoma (MIA), and invasive adenocarcinoma (IAC). The 8th edition (2015) of the tumor, node, metastasis (TNM) staging system was used to determine the disease stage [10]. The high-throughput 56G sequencing panel was used for gene sequencing.

### Statistical analysis

All the data were analyzed by means of SPSS 22.0 (SPSS Inc., Chicago, IL, USA). The normally distributed measurements were expressed by mean ± standard deviation (x ± s), and *T* test was used to compare differences between groups. The non-normally distributed measurements were analyzed by Wilcoxon rank-sum test. The enumeration data were expressed by constituent ratio and percentage and analyzed by Chi-square test. Fisher's exact test was used as an alternative to the fourfold table Chi-square test if any of the expected frequencies is less than 5. The *P* value of less than 0.05 indicates that the difference is statistically significant.

## Results

### Patients’ baseline data

From 2009 to 2021, 2,092 patients with lung adenocarcinoma manifesting as GGNs on HRCT images in the West China Hospital of Sichuan University were collected. According to the above-mentioned inclusion and exclusion criteria, a total of 1,800 cases (545 males and 1,255 females) were finally included in this study. Among them, 285 individuals (269 males and 16 females) had a smoking history. The mean age at diagnosis was 54.78 (23–84). In the light of the definition of individuals at high risk for lung cancer in the Chinese Expert Consensus on Screening and Management of Lung Cancer [6], all the cases were divided into high-risk group and non-high-risk group (509 vs 1,291). The former was dominated by male smokers, and the latter by female non-smokers. There were statistical differences between the two groups in gender, smoking history, medical history, family history of tumors, and exposure history (all *P* < 0.05), in line with the meaning of grouping. In terms of detection methods, which encompass physical examination as well as various other approaches, our analysis revealed no statistically significant difference between the two groups (*P* > 0.05). According to the 8th edition (2015) of the TNM staging system [10], all these lung adenocarcinomas included were composed of 1,600 (88.89%) stage IA, 188 (10.44%) stage IB, 8 (0.44%) stage IIB, 2 (0.11%) stage IIIA, 1 (0.06%) stage IIIB, and 1 (0.06%) stage IVA, and the latter four cases were advanced (see Additional file 1: Figure S1 for details). No statistical difference was found in clinical staging between the two groups (*P* = 0.488). According to the 2021 WHO Classification of Lung Tumors [9], 1,237 patients were classified into precursor lesions (AAH + AIS) (157, 12.70%), minimally invasive adenocarcinoma (MIA) (504, 40.74%), and invasive adenocarcinoma (IAC) (576, 46.56%). The frequency of IAC was higher in the high-risk group (*P* = 0.001). The preoperative follow-up time in the high-risk group was relatively longer, especially the follow-up lasting longer than 12 months (*P* = 0.015) (Table 1 and Additional file 2: Table S1).

**Table 1:** Baseline data of risk groups

	High risk (509)	Non-high risk (1291)	*P *value
Gender			< 0.001*
Male	288 (56.58)	257 (19.91)	
Female	221 (43.42)	1034 (80.09)	
Age			< 0.001*
Mean ± SD	57.58 ± 9.262	53.67 ± 11.590	
Smoking history			
Yes	265 (52.06)	20 (1.55)	< 0.001*
No	244 (47.94)	1271 (98.45)	
Medical history			
Tumor	78 (15.32)	6 (0.46)	< 0.001*
Pulmonary fibrosis	1 (0.20)	0	0.283
COPD	4 (0.79)	0	0.006*
Pulmonary tuberculosis	11 (2.16)	2 (0.15)	< 0.001*
Family history of tumors			
Lung cancer	192 (37.72)	16 (1.24)	< 0.001*
Other tumors	80 (15.72)	122 (9.45)	< 0.001*
Exposure history			
Oil, smoke and dust	9 (1.77)	1 (0.08)	< 0.001*
Asbestos, beryllium, uranium, radon, etc	1 (0.20)	1 (0.08)	0.486
GGN detection way			0.413
Physical examination	302 (59.33)	793 (61.43)	
Others	207 (40.67)	498 (38.57)	
Clinical stage			0.488
IA	457 (89.78)	1143 (88.54)	
IB	48 (9.43)	140 (10.84)	
IIB	3 (0.59)	5 (0.39)	
IIIA	0	2 (0.15)	
IIIB	1 (0.20)	0	
IV	0	1 (0.08)	
Pathological subtype^c^ (total = 1237)			< 0.001*
AAH + AIS	29 (8.43)	128 (14.33)	
MIA	123 (35.76)	381 (42.67)	
IAC	192 (55.81)	384 (43.00)	
Time from detection to diagnosis			0.015*
≤ 3 months	242 (47.54)	629 (48.72)	
4–12 months	151 (29.67)	440 (34.08)	
> 12 months	116 (22.79)	222 (17.2)	

^*^P < 0.05 or *P* < 0.01 after Bonferroni correction is considered statistically different

^c^1237/1800 cases were classified pathologically according to the existing pathological subtypes

### Imaging features of risk groups

There were 115 (22.59%) pGGNs and 394 (77.41%) mGGNs in the high-risk group, and 280 (21.69%) and 1,011 (78.31%) in the non-high-risk group, with no statistical difference. All the GGNs were classified into three categories by size (≤ 10 mm, 11–20 mm and 21–30 mm). The average nodule diameters in the non-high-risk and high-risk groups were 13.6 mm and 15.1 mm, respectively. In the high-risk group, GGNs with the diameters of no more than 10 mm and 21–30 mm accounted for 40.36% and 14.95%, respectively, significantly different from those in the non-high-risk group (*P* < 0.001). Most of the GGNs were observed in the superior lobes of both lungs, especially the right one (*P* = 0.040). No statistical difference was found in imaging signs between groups (*P* = 0.648) (Table 2).

**Table 2:** Imaging features of risk groups

	High risk (509)	Non-high risk (1291)	*P *value
Density			**0.676**
pGGN	115 (22.59)	280 (21.69)	
mGGN	394 (77.41)	1011 (78.31)	
Size (mm)			
Average	15.1	13.6	< 0.001*
Grouping by size			< 0.001*
≤ 10	158 (31.04)	521 (40.36)	
10–20	243 (47.74)	577 (44.69)	
20–30	108 (21.22)	193 (14.95)	
Location			0.040*
Right superior lobe	208 (40.86)	501 (38.81)	
Right middle lobe	31 (6.09)	81 (6.27)	
Right inferior lobe	65 (12.77)	209 (16.19)	
Left superior lobe	155 (30.45)	329 (25.48)	
Left inferior lobe	50 (9.82)	171 (13.25)	
Imaging sign			
Lobulation	105 (20.63)	254 (19.67)	0.648
Vacuole	89 (17.49)	185 (14.33)	0.093
Spiculation	108 (21.22)	225 (17.43)	0.062
Calcification	3 (0.59)	6 (0.46)	0.718
Air bronchogram	17 (3.34)	43 (3.33)	0.992
Pleural indentation	88 (17.29)	212 (16.42)	0.657
Vessel convergence	17 (3.34)	35 (2.71)	0.473

^*^P < 0.05 or *P* < 0.01 after Bonferroni correction is considered statistically different

### Gene expression of risk groups

**Fig. 1:**
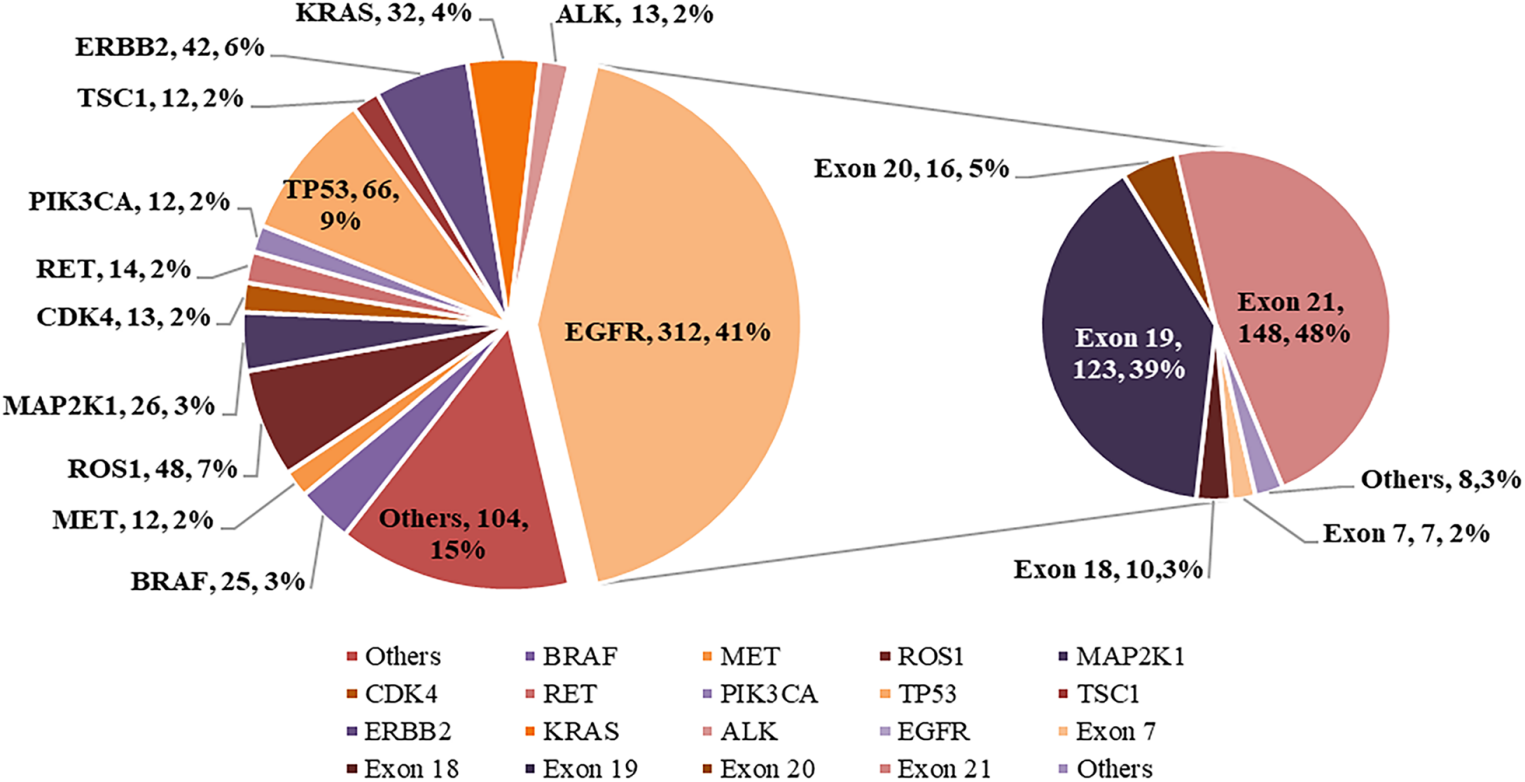
G56 sequencing results of all cases

**Fig. 2:**
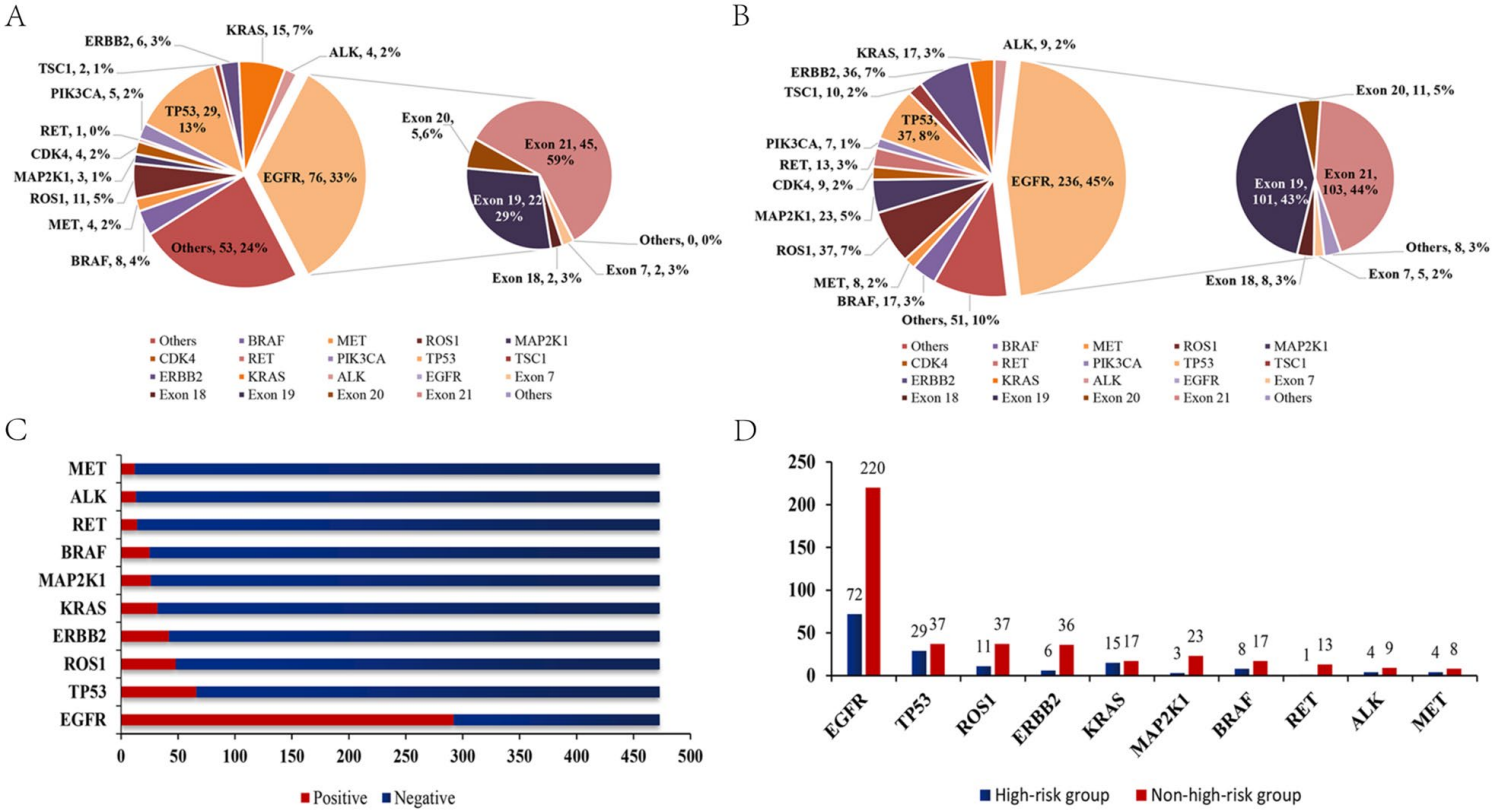
Distribution of genes among different risk groups. **A** G56 sequencing results of the high-risk group. **B** G56 sequencing results of the non-high-risk group. **C** Top 10 gene mutations of all case. **D** Top 10 gene mutations of risk groups

A total of 473 patients underwent high-throughput 56G sequencing, and 711 genes (incl. 217 in 115 high-risk cases and 494 in 358 non-high-risk cases) were tested positive for mutation. Among the 473 cases, the most common mutations were observed in *EGFR* (292, 61.73%), *TP53* (66, 13.95%) and *ROS1* (48, 10.15%). Among all the positive genes, *EGFR*, *TP53* and *ROS1* accounted for 41.07%, 9.28% and 6.75%, respectively. A total of 312 positive loci were observed in *EGFR* + cases, 87% of which were located in exons 19 and 21 (Fig. 1). *EGFR* mutation accounted for 62.61% (72/115) in the high-risk group and 61.45% (220/358) in the non-high-risk group, with no statistical difference (*P* = 0.824). *TP53* and *KRAS* mutations accounted for 25.22% (29/115) and 13.04% (15/115) in the high-risk group, significantly higher than 10.34% (37/358) and 4.75% (17/358) in the non-high-risk group (*P* < 0.050). (Table 3 and Fig. 2).

**Table 3:** Top 10 genes positive for mutation of risk groups

Top 10 genes	High risk (*N* = 115, % among the total cases)	Non-high-risk (*N* = 358, % among the total cases)	*P *value
EGFR	72 (62.61)	220 (61.45)	0.824
TP53	29 (25.22)	37 (10.34)	< 0.001*
ROS1	11 (9.57)	37 (10.34)	0.812
ERBB2	6 (5.22)	36 (10.06)	0.113
KRAS	15 (13.04)	17 (4.75)	0.002*
ALK	4 (3.48)	9 (2.51)	0.527
MET	4 (3.48)	8 (2.23)	0.497
RET	1 (0.87)	13 (3.63)	0.204
BRAF	8 (6.96)	17 (4.75)	0.357
MAP2K1	3 (2.61)	23 (6.42)	0.158

^*^: *P* < 0.05 or *P* < 0.01 after Bonferroni correction is considered statistically different

### Correlation between *EGFR*, *TP53* and *KRAS* mutations and clinical characteristics

It was found that the included *EGFR* mutations were not correlated with gender and smoking history (both *P* > 0.05). Considering the age of individuals at high risk for lung cancer, the incidence of *EGFR* mutation in patients aged 40 and older was higher than that in those under age 40 (*P* < 0.001). In the patients with *EGFR* mutation, the ratio of mutant in stage IA was significantly lower than that of wild type (91.10% vs 97.24%,* P* = 0.019). In terms of pathological classification, mutant *EGFR* had a higher prevalence in IAC (69.6%), while wild-type *EGFR* had a higher prevalence in MIA (53.42%) and earlier-stage subtypes (*P* < 0.001). The incidences of *TP53* and *KRAS* mutations were higher in male smokers than those in female non-smokers (both *P* < 0.05). The clinical staging and pathological classification of *TP53* + patients were later than those of the wild type (*P* < 0.05). No statistical difference was found in the clinical staging and pathological classification between mutant and wild-type *KRAS* genes. No statistical difference was found in *TP53* and *KRAS* mutations between age groups (both *P* > 0.05). (see Additional file 3: Table S2).

### Correlation between *EGFR–TP53* mutant combinations and the clinical, pathological and imaging features and risk grouping of patients

*TP53* co-mutation was observed in 55/292 (18.84%) *EGFR* + patients included in this study. The mean age of *EGFR* + /*TP53* + group was significantly higher than that of *EGFR*-/*TP53*- group (*P* < 0.001). There were statistical differences in mutant combinations of *EGFR* and *TP53* between males and females (*P* < 0.001). In the *EGFR* + /*TP53* + and *EGFR*-/*TP53* + groups, the proportion of males was close to or larger than that of females; while in the other two (*EGFR* + /*TP53*- and *EGFR*-/*TP53*-) groups, females were in a majority. There were also statistical differences in the mutant combinations between smokers and non-smokers (*P* < 0.001). The proportion of smokers in *EGFR*-/*TP53* + group was the largest (81.82%). Besides, statistical differences were found in two imaging signs, spiculation and pleural indentation (all *P* < 0.05). Statistical differences were also found in clinical staging (all *P* < 0.05). The proportion of patients in stage IA in *EGFR* + /*TP53* + group was significantly lower than those in *EGFR* + /*TP53*- and *EGFR*-/*TP53*- groups (*P* < 0.05). The proportion of patients in stage IB in *EGFR* + /*TP53* + group was significantly higher than those in *EGFR* + /*TP53*- and *EGFR*-/*TP53*- groups (*P* < 0.05). However, no correlation was found between clinical staging and the rest *EGFR*-/*TP53* + group. In terms of pathological classification, no statistical difference was found in AAH + AIS. The incidence of *EGFR*-/*TP53*- group in MIA was significantly higher than those of *EGFR* + /*TP53* + and *EGFR* + /*TP53*- groups, while the incidence of *EGFR*-/*TP53*- group in IAC was significantly lower than those of the other three groups, indicating statistical differences (all *P* < 0.05). Besides, no correlation was found between the mutant combinations and the risk grouping (Table 4).

**Table 4:** Correlation between EGFR–TP53 mutant combinations and the clinical, pathological and imaging features and risk grouping of patients

	EGFR + /TP53 + N = 55	EGFR + /TP53- N = 237	EGFR-/TP53- N = 170	EGFR-/TP53 + N = 11	*P *value
Age					< 0.001*
	57.42 ± 9.473 a	54.34 ± 11.545 a	51.06 ± 11.286 b	58.36 ± 9.244 a	
Gender					< 0.001*
Male	27 (49.09)a	70 (29.54)b	47 (27.65)b	9 (81.82)a	
Female	28 (50.91)a	167 (70.46)b	123 (72.35)b	2 (18.18)a	
Smoking history					< 0.001*
Yes	9 (16.36)a	31 (13.08)a	21 (12.35)a	9 (81.82)b	
No	46 (83.64)a	206 (86.92)a	149 (87.65)a	2 (18.18)b	
Family history of lung cancer					0.571
Yes	5 (9.09)a	22 (9.28)a	20 (11.76)a	0 (0.00)a	
No	50 (90.91)a	215 (90.72)a	150 (88.23)a	11 (100.00)a	
Family history of other tumors					0.644
Yes	7 (12.73)a	26 (10.97)a	17 (10.00)a	0 (0.00)a	
No	48 (87.27)a	211 (89.03)a	153 (90.00)a	11 (100.00)a	
Nodule location					0.109
Right superior lobe	22 (40.00)a,b	84 (35.44)b	63 (37.06)b	9 (81.82)a	
Right middle lobe	3 (5.45)a	14 (5.91)a	8 (4.71)a	0 (0.00)a	
Right inferior lobe	6 (10.91)a	46 (19.41)a	26 (15.29)a	0 (0.00)a	
Left superior lobe	13 (23.64)a	64 (27.00)a	56 (32.94)a	2 (18.18)a	
Left inferior lobe	11 (20.00)a	29 (12.24)a	17 (10.00)a	0 (0.00)a	
Imaging sign					
Lobulation	16 (29.09)a	50 (21.10)a	27 (15.88)a	2 (18.18)a	0.185
Vacuole	9 (16.36)a	37 (15.61)a	29 (17.06)a	1 (9.09)a	0.905
Spiculation	13 (23.64)a	42 (17.72)a	14 (8.24)b	2 (18.18)a,b	0.013*
Calcification	0 (0.00)a	2 (0.84)a	3 (1.76)a	0 (0.00)a	0.654
Air bronchogram	1 (1.82)a	7 (2.95)a	4 (2.35)a	0 (0.00)a	0.899
Pleural indentation	16 (29.09)a	54 (22.78)a	16 (9.41)b	1 (9.09)a,b	0.001*
Vessel convergence	2 (3.64)a	9 (3.80)a	6 (3.53)a	0 (0.00)a	0.509
Clinical stage					0.020*
IA	40 (72.73)a	211 (89.03)b	155 (91.18)b	10 (90.91)a,b	
IB	13 (23.64)a	24 (10.13)b	14 (8.24)b	1 (9.09)a,b	
IIB	2 (3.64)a	2 (0.84)a	1 (0.59)a	0 (0.00)a	
Pathological subtype ^c^ (*n* = 411)					< 0.001*
AAH + AIS	0 (0.00)a	6 (2.96)a	10 (6.67)a	0 (0.00)a	
MIA	7 (14.89)a	63 (31.03)a	84 (56.00)b	2 (18.18)a,b	
IAC	40 (85.11)a	134 (66.01)a	56 (37.33)b	9 (81.82)a	
Risk grouping					0.081
High risk	20 (36.36)a	52 (21.94)a	34 (20.00)a	2 (18.18)a	
Non-high risk	35 (63.64)a	185 (78.06)a	136 (80.00)a	9 (81.82)a	

The same subscript letter indicates the difference between mutant combinations is not statistically significant (*P* > 0.05), while different subscripts indicate the difference between mutant combinations is statistically significant (*P* < 0.05). Age is expressed as Mean ± SD and the rest is expressed as frequency (percentage)

^*^P < 0.05 or *P* < 0.01 after Bonferroni correction is considered statistically different

^c^411/473 cases were classified pathologically according to the existing pathological subtypes

## Discussion

The progress and popularization of imaging technology effectively promote the detection and treatment of pulmonary GGNs [11, 12]. Pulmonary GGNs can be observed in inflammation, hemorrhage, and even early stage tumors, some of them are temporary; however, if they persist for more than 3 months, they cannot be excluded as early stage lung adenocarcinoma [13]. Compared with solid nodules, GGN-featured lung adenocarcinoma has an indolent course following the natural progression of AAH–AIS–MIA–IAC. Its volume doubling time (VDT) ranges from 759 to 1832 days. Lengthy VDT is associated with better prognosis and lower invasion [14]. In the 2021 WHO Classification of Lung Tumors, AIS has been added to the group of glandular precursor lesions along with AAH [9]. In this study, the frequency of AAH + AIS totaled 12.70%, and that in the non-high-risk group was higher than that in the high-risk group (14.33% vs 8.43%, *P* < 0.001). Heidinger et al. recommended a 36-month follow-up for both single and multiple pulmonary GGNs [15]. However, in this study, the number of patients with a follow-up of less than 3 months accounted for nearly 50% of the total and in both high-risk and non-high-risk groups. Only 18.78% were followed up for more than 12 months. Few patients were followed up for more than 36 months. The time from detection to diagnosis in the non-high-risk group was even shorter than that in the high-risk group. It can be seen that patients at non-high-risk level may experience more stress caused by their living environment and anxiety, uncertainty about the progress of nodules, as well as the impact of surgery on lung function. Although GGNs can be diagnosed and treated at an early stage, the advantages and disadvantages of indolent tumor surgery still need further verification. Hence, efforts should be made to figure out more scientific management, follow-up and treatment strategies, so as to improve the survival and quality of life of patients while avoiding excessive diagnosis and treatment and relieving their psychological burden [16, 17].

In Europe and the US, individuals at high risk for lung cancer are defined as those aged 50 and older who have a 30 pack-year smoking history [18]. Given the domestic conditions, individuals at high risk for lung cancer in China was defined in 2019 [6] as those aged 40 and older who have a 20 pack- or 400 cigarette-year smoking history and currently smoke or have quit within the past 15 years; have a history of environmental or occupational exposure (such as asbestos, beryllium, uranium, radon, etc.); are complicated with chronic obstructive pulmonary disease or diffuse pulmonary fibrosis, or have a history of pulmonary tuberculosis; or have a history of malignant tumor or a family history of lung cancer. Plenty of domestic and foreign researches showed that LDCT screening for people at high risk could effectively detect early stage lung cancers and reduce their mortality [19, 20]. Since 2005, a number of earlier screening and treatment programs have been carried out in China, effectively encouraging public participation in lung cancer screening and earlier diagnosis [21, 22]. In this study, 1,800 patients with GGN-featured lung adenocarcinoma detected in our hospital from 2009 to 2021, divided into high-risk group and non-high-risk group, were analyzed statistically. The high-risk group was dominated by male smokers, while the non-high-risk group by female non-smokers. As a whole, more women than men were included in this study (69.72% vs 30.28%), consistent with previous reports on the predominance of female non-smokers in lung adenocarcinoma [23, 24]. In this study, 60.83% of the total patients were detected during their physical examination, and the proportion in the high-risk group was slightly lower than that in the non-high-risk group [302 (59.33%) vs 793 (61.43%)], without no significant difference. However, it also suggests: (1) due to the rising incidence of lung cancer among non-high-risk individuals, screening for non-high-risk individuals should not be ignored [25, 26] and the current screening criteria for people at high risk for lung cancer in China may not be applicable to the screening of early stage lung cancer manifesting as GGNs. (2) Additional attentions should be paid to the popularization of physical examination and screening, especially among people at high risk for lung cancer. In addition, no significant difference was founded in clinical staging between groups (*P* = 0.488). For pathological classification, the frequency of IAC in the high-risk group was higher than that in the non-high-risk group (55.81% vs 43.00%), while the frequency of precursor lesions (AAH + AIS) in the high-risk group was lower than that in the non-high-risk group (8.43% vs 14.33%), indicating significant differences (both *P* < 0.001). It can be seen that, the high-risk factors for lung cancer are also for the progression of tumors.

In terms of gene expression, the incidence of *EGFR* mutation in Asian population is more than a half, significantly higher than that in Europe and the US, and occurs predominantly in exons 19 and 21 [2, 27]. In this study, the incidence reached 61.73%, 87% of which occurred in exons 19 and 21, consistent with the above-mentioned research results. It was reported that *EGFR* mutation tended to occur in female non-smokers [28, 29]. In this study, no significant difference was found in *EGFR* mutation in terms of gender, smoking history and risk grouping, which may be due to the difference between early stage lung adenocarcinoma and middle- and advanced-stage lung adenocarcinomas. A previous comparative analysis between advanced- and early stage lung adenocarcinomas also confirmed that *EGFR* mutation was not correlated with gender, but the proportion of smokers in patients with early stage lung adenocarcinoma was higher than that in the advanced [30]. Ren et al. also stated that *EGFR* mutation was not correlated with gender [31]. Besides, we found that there was no statistical difference in the detection rate of *EGFR* + patients between the high-risk group and the non-high-risk group (*P* = 0.824). The *TP53* gene is the most frequently mutated gene across all cancer types [32]. Mutant *TP53* endows cancer cells with more malignant, playing an important role in therapy resistance and poor prognosis [33, 34]. Previous researches showed that the incidence of *TP53* mutations varied greatly in non-small cell lung cancer and was correlated with the pathological subtypes, reaching 81% in lung squamous cell carcinoma, 46% in lung adenocarcinoma [35], and 90% in small cell lung cancer [36]. In this study, even in patients with early stage lung adenocarcinoma, the incidence of *TP53* mutations was as high as 13.95%. In addition, cigarette smoking produces a heavy burden of *TP53* mutations [37]. In this study, the proportions of smokers and *TP53* mutations in the high-risk group were significantly higher than those in the non-high-risk group (both *P* < 0.001). The *KRAS* gene is a member of the rat sarcoma viral oncogene family (*RAS*), and the incidence of *KRAS* mutation in Asia is 5–15% [38]. In this study, the incidence of *KARS* mutation was 6.76%. There is, however, no consensus on the impact of cigarette smoking on the frequency in *KAR*S mutations [39, 40]. Choi et al. found that *KRAS* mutation was common in male smoker and associated with invasive mucinous adenocarcinoma on histologic analysis [41]. In this study, the incidence of *KARS* mutation in the high-risk group dominated by male smokers was higher than that in the non-high-risk group (*P* < 0.001), indicating the impact of cigarette smoking on the frequency of *KARS* mutations.

Targeted therapy renewed the treatment mode for lung cancers. However, for concurrent *TP53* mutations in *EGFR* mutated lung cancers, the prognosis may be affected by the poor effect of targeted therapy [33, 42, 43]. According to previous researches, concurrent *TP53* mutations were very common in *EGFR* mutated lung adenocarcinoma with an incidence of 54.6–64.6% [44], which is inconsistent with the incidence of 18.84% in this study. This may be because most of the cases in this study are early stage lung cancers manifesting as GGNs. In this study, the proportion of males in the *EGFR* + /*TP53* + group was higher than those in the *EGFR* + /*TP53*- and *EGFR*-/*TP53*- groups. The proportion of patients with clinical stage IA lung adenocarcinoma in the *EGFR* + /*TP53* + group was significantly lower than those in the *EGFR* + /*TP53*- and *EGFR*-/*TP53*- groups. The frequency of IAC in the *EGFR* + /*TP53* + group was higher than those in the other groups. It suggests that *EGFR* + /*TP53* + is prone to promote the growth, invasion and metastasis of tumor cells [45, 46], thus affecting the survival and prognosis of patients with tyrosine kinase inhibitor (TKI) treatment. For patients with GGN-featured lung cancer, a follow-up after surgery rather than targeted treatment is recommended. However, some patients suffered a recurrence of multiple nodules after surgery. Therefore, the necessity of targeted treatment after surrey for GGN-featured lung cancers complicated with gene mutations is worth of further study.

## Conclusions

Screening for non-high-risk individuals should not be ignored due to the rising incidence of early stage lung cancers manifesting as GGNs among non-high-risk individuals. Besides, the existing screening criteria for people at high risk for lung cancer in China may not be applicable to the screening of early stage lung cancers manifesting as GGNs. The popularization of physical examination among people at high risk for lung cancer in China is still insufficient, and the follow-up time for patients with GGNs should be extended. Efforts should be made to figure out more scientific management, follow-up and treatment strategies. Although similar to that in lung adenocarcinoma, gene mutations in GGN-featured lung cancers still have their own characteristics. The necessity of targeted treatment after surrey for GGN-featured lung cancers complicated with gene mutations is worth of further study.

### Supplementary Information

**Additional file 1: Figure S1.** Images of Four Advanced GGN-featured Lung Cancers. **A** Cough for more than 2 months, an IAC observed in the superior lobe of the right lung, pleural involvement, mediastinal lymph node metastasis, stage IIIA. **B** Detected for more than 2 months via physical examination, an IAC observed in the superior lobe of the right lung, pleural involvement, mediastinal and supracarinal lymph node metastases, stage IIIB. **C** Detected for more than 1 month via physical examination, an IAC observed in the inferior lobe of the left lung, invading the pleura, mediastinal lymph node metastasis, stage IIIA. **D** Detected for 6 days via physical examination, an IAC observed in the superior lobe of the right lung, malignant nodules observed in the pleura, no lymph node metastasis, stage IVA.

**Additional file 2: Table S1.** Patients’ baseline data.

**Additional file 3: Table S2.** Correlation between EGFR, TP53 and KRAS mutations and clinical characteristics.

## Data Availability

The data used and analyzed in this study can be obtained from the corresponding author on reasonable request.

## Abbreviations

GGNs: Ground glass nodules
HRCT: High-resolution computed tomography
LDCT: Low-dose computed tomography
PACS: Picture archiving and communication systems
pGGN: Pure ground-glass nodule
mGGN: Mixed ground-glass nodule
AAH: Atypical adenomatous hyperplasia
AIS: Adenocarcinoma in situ
MIA: Minimally invasive adenocarcinoma
IAC: Invasive adenocarcinoma
TNM: Tumor, node, metastasis
VDT: Volume doubling time
TKI: Tyrosine kinase inhibitor
