# Supplementary figures and images for: Correlation analysis of clinical, pathological, imaging and genetic features of ground-glass nodule featured lung adenocarcinomas between high-risk and non-high-risk individuals
Source: Eur J Med Res. 2023 Nov 4;28:478. doi: 10.1186/s40001-023-01462-3 (PMC10625210; doi:10.1186/s40001-023-01462-3)

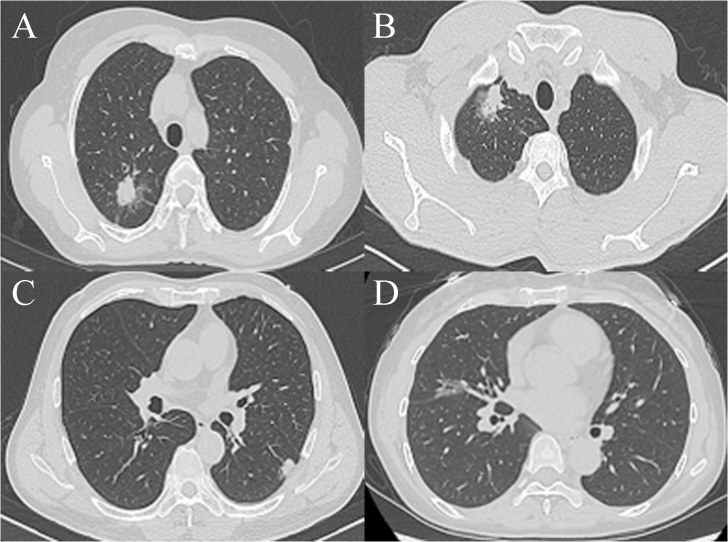

Supplement: Supplementary file 1 — Additional file 1: Figure S1. Images of Four Advanced GGN-featured Lung Cancers. A Cough for more than 2 months, an IAC observed in the superior lobe of the right lung, pleural involvement, mediastinal lymph node metastasis, stage IIIA. B Detected for more than 2 months via physical examination, an IAC observed in the superior lobe of the right lung, pleural involvement, mediastinal and supracarinal lymph node metastases, stage IIIB. C Detected for more than 1 month via physical examination, an IAC observed in the inferior lobe of the left lung, invading the pleura, mediastinal lymph node metastasis, stage IIIA. D Detected for 6 days via physical examination, an IAC observed in the superior lobe of the right lung, malignant nodules observed in the pleura, no lymph node metastasis, stage IVA. [file 40001_2023_1462_MOESM1_ESM.tif]
