# Supplementary material for: Correlation analysis of clinical, pathological, imaging and genetic features of ground-glass nodule featured lung adenocarcinomas between high-risk and non-high-risk individuals
Source: Eur J Med Res. 2023 Nov 4;28:478. doi: 10.1186/s40001-023-01462-3 (PMC10625210; doi:10.1186/s40001-023-01462-3)
Supplement: Supplementary file 2 — Additional file 2: Table S1. Patients’ baseline data. [file 40001_2023_1462_MOESM2_ESM.docx]

**Additional file Tab.1** Patients’ Baseline Data

|  | **Number (%), total=1800** |
| --- | --- |
| **Risk grouping** |  |
| High-risk | 509 (28.28) |
| Non-high-risk | 1291 (71.72) |
| **Gender** |  |
| Male | 545 (30.28) |
| Female | 1255 (69.72) |
| **Age** |  |
| Mean | 54.78 |
| **Smoking history** |  |
| Yes | 285 (15.83) |
| No | 1515 (84.17) |
| **Medical history** |  |
| Tumor | 84 (4.67) |
| Pulmonary fibrosis | 1 (0.06) |
| COPD | 4 (0.22) |
| Pulmonary tuberculosis | 13 (0.72) |
| **Family history of tumors** |  |
| Lung cancer | 208 (11.56) |
| Other tumors | 202 (11.22) |
| **Exposure history** |  |
| Oil, smoke and dust | 10 (0.56) |
| Asbestos, beryllium, uranium, radon, etc. | 2 (0.11) |
| **GGN detection way** |  |
| Physical Examination | 1095 (60.83) |
| Others | 705 (39.17) |
| **Time from detection to diagnosis (months)** |  |
| Mean | 9.59 |
| ≤3 months | 871 (48.39) |
| 4-12 months | 591 (32.83) |
| >12 months | 338 (18.78) |
| **Clinical stage** |  |
| IA | 1600 (88.89) |
| IB | 188 (10.44) |
| IIB | 8 (0.44) |
| IIIA | 2 (0.11) |
| IIIB | 1 (0.06) |
| IVA | 1 (0.06) |
| **Pathological subtype ^c^ (total=1237)** |  |
| AAH+AIS | 157 (12.70) |
| MIA | 504 (40.74) |
| IA | 576 (46.56) |
| **Nodule Size (mm)** |  |
| ≤10 | 679 (37.72) |
| 10-20 | 820 (45.56) |
| 20-30 | 301 (16.72) |
| **Density** |  |
| pGGN | 395 (21.94) |
| mGGN | 1405 (78.06) |
| **Gene positive for mutation ^d^ (N=473)** |  |
| EGFR | 292 (61.73) |
| TP53 | 66 (13.95) |
| ROS1 | 48 (10.15) |
| ERBB2 | 42 (8.88) |
| KRAS | 32 (6.77) |
| MAP2K1 | 26 (5.50) |
| BRAF | 25 (5.28) |
| RET | 14 (2.96) |
| ALK | 13 (2.75) |
| MET | 12 (2.54) |

c: 1237/1800 cases had clear pathological subtypes.

d: 473/1800 cases underwent G56 sequencing.
