# Supplementary material for: Correlation analysis of clinical, pathological, imaging and genetic features of ground-glass nodule featured lung adenocarcinomas between high-risk and non-high-risk individuals
Source: Eur J Med Res. 2023 Nov 4;28:478. doi: 10.1186/s40001-023-01462-3 (PMC10625210; doi:10.1186/s40001-023-01462-3)
Supplement: Supplementary file 3 — Additional file 3: Table S2. Correlation between EGFR, TP53 and KRAS mutations and clinical characteristics. [file 40001_2023_1462_MOESM3_ESM.docx]

**Additional Tab.2** Correlation between EGFR, TP53 and KRAS Mutations and Clinical Characteristics

| **Clinical Factor** |  | **EGFR** | | ***P-*value** | **TP53** | | ***P-*value** | **KRAS** | | ***P-*value** |
| --- | --- | --- | --- | --- | --- | --- | --- | --- | --- | --- |
|  | **N (%)** | **Mutation**  **N=292，61.73%** | **Wild type**  **N=181，38.27%** |  | **Mutation**  **N=66，13.95%** | **Wild type**  **N=407，86.05%** |  | **Mutation**  **N=32，6.77%** | **Wild type**  **N=441，93.23%** |  |
| **Gender** |  |  |  | 0.606 |  |  | ***＜0.001**** |  |  | ***0.003**** |
| Male | 153 (32.35) | 97 (33.22) | 56 (30.94) |  | 36 (54.55) | 117 (28.75) |  | 18 (56.25) | 135 (30.61) |  |
| Female | 320 (67.65) | 195 (66.78) | 125 (69.06) |  | 30 (45.45) | 290 (71.25) |  | 14 (43.75) | 306 (69.39) |  |
| **Age** |  |  |  | ***＜0.001**** |  |  | 0.176 |  |  | 0.060 |
| <40 | 45 (9.51) | 15 (5.14) | 30 (16.57) |  | 3 (4.55) | 42 (10.32) |  | 0 | 45 (10.20) |  |
| ≥40 | 428 (90.49) | 277 (94.86) | 151 (83.43) |  | 63 (95.45) | 365 (89.68) |  | 32 (100.00) | 396 (89.80) |  |
| **Smoking history** |  |  |  |  |  |  |  |  |  |  |
| Yes | 70 (14.80) | 40 (13.70) | 30 (16.57) | 0.392 | 18 (27.27) | 52 (12.78) | ***0.002**** | 12 (37.50) | 58 (13.15) | ***＜0.001**** |
| No | 403 (85.20) | 252 (86.30) | 151 (83.43) |  | 48 (72.73) | 355 (87.22) |  | 20 (62.50) | 383 (86.85) |  |
| **Clinical stage** |  |  |  | ***0.019**** |  |  | ***0.003**** |  |  | 0.068 |
| IA | 442 (93.45) | 266 (91.10) | 176 (97.24) |  | 55 (83.33) | 387 (95.08) |  | 27 (84.38) | 415 (94.10) |  |
| IB | 27 (5.71) | 23 (7.88) | 4 (2.21) |  | 10 (15.15) | 17 (4.18) |  | 4 (12.50) | 23 (5.22) |  |
| IIA | 1 (0.21) | 1 (0.34) | 0 |  | 0 | 1 (0.25) |  | 0 | 1 (0.23) |  |
| IIB | 3 (0.63) | 2 (0.68) | 1 (0.55) |  | 1 (1.52) | 2 (0.49) |  | 1 (3.12) | 2 (0.45) |  |
| **Pathological subtype ^c^**  **(total=411)** |  |  |  | ***＜0.001**** |  |  | ***0.001**** |  |  | 0.143 |
| AAH+AIS | 16 (3.89) | 6 (2.40) | 10 (6.21) |  | 0 | 16 (4.53) |  | 3 (11.11) | 13 (3.39) |  |
| MIA | 156 (37.96) | 70 (28.00) | 86 (53.42) |  | 9 (15.52) | 147 (41.64) |  | 10 (37.04) | 146 (38.02) |  |
| IAC | 239 (58.15) | 174 (69.60) | 65 (40.37) |  | 49 (84.48) | 190 (53.83) |  | 14 (51.85) | 225 (58.59) |  |

*: *P*<0.05 or *P*<0.01 after Bonferroni correction is considered statistically different.

c: 411/473 cases had clear pathological subtypes.
